# The anguibactin biosynthesis and transport genes are encoded in the chromosome of *Vibrio harveyi*: a possible evolutionary origin for the pJM1 plasmid–encoded system of *Vibrio anguillarum*?

**DOI:** 10.1002/mbo3.65

**Published:** 2013-01-18

**Authors:** Hiroaki Naka, Luis A Actis, Jorge H Crosa

**Affiliations:** 1 Department of Molecular Microbiology and Immunology, Oregon Health and Science University3181 SW Sam Jackson Park Road, Portland, Oregon, 97239; 2 Department of Microbiology, Miami University, Oxford32 Pearson Hall, Oxford, Ohio, 45056

**Keywords:** Anguibactin, iron transport, siderophore, *Vibrio anguillarum*, *Vibrio harveyi*

## Abstract

Many *Vibrio anguillarum* serotype O1 strains carry 65-kb pJM1-type plasmids harboring genes involved in siderophore anguibactin biosynthesis and transport. The anguibactin system is an essential factor for *V. anguillarum* to survive under iron-limiting conditions, and as a consequence, it is a very important virulence factor of this bacterium. Our comparative analysis of genomic data identified a cluster harboring homologs of anguibactin biosynthesis and transport genes in the chromosome of *Vibrio harveyi*. We have purified the putative anguibactin siderophore and demonstrated that it is indeed anguibactin by mass spectrometry and specific bioassays. Furthermore, we characterized two genes, *angR* and *fatA*, in this chromosome cluster that, respectively, participate in anguibactin biosynthesis and transport as determined by mutagenesis analysis. Furthermore, we found that the *V. harveyi* FatA protein is located in the outer membrane fractions as previously demonstrated in *V. anguillarum*. Based on our data, we propose that the anguibactin biosynthesis and transport cluster in the *V. anguillarum* pJM1 plasmid have likely evolved from the chromosome cluster of *V. harveyi* or vice versa.

## Introduction

Iron is an essential element for nearly all living organisms as it is involved in many metabolic processes; however, the amount of free iron in the environment as well as in the host is very limited due to its insolubility at neutral pH in the presence of oxygen and chelation by high-affinity iron-binding host products. To overcome these iron-limiting conditions, bacteria express high-affinity iron acquisition systems. One of them is siderophore-mediated iron transport system. Bacteria biosynthesize and secrete siderophores that chelate ferric iron and the ferric-siderophore complex are then taken up into the bacterial cytosol via specific outer membrane receptors and ATPase-dependent ABC transporters or proton motive force–dependent permeases (Crosa and Walsh 2002; Cuiv et al. 2004; Raymond and Dertz 2004; Winkelmann 2004; Hannauer et al. 2010; Reimmann 2012).

*Vibrio anguillarum* is a marine pathogen that causes serious hemorrhagic septicemia in wild and cultured fish (Actis et al. 2011; Naka and Crosa 2011). Many *V. anguillarum* serotype O1 strains produce the siderophore anguibactin and take up ferric-anguibactin via the cognate outer membrane receptor FatA (Actis et al. 2011; Naka and Crosa 2011). The anguibactin-mediated system is an essential factor for the multiplication of this bacterium under iron-limiting conditions, and it is the most important virulence factor of this fish pathogen (Crosa 1980; Actis et al. 2011; Naka and Crosa 2011). The majority of genes involved in anguibactin biosynthesis and transport are encoded in the 65-kb pJM1 or pJM1-like plasmids of *V. anguillarum* serotype O1 strains (Crosa and Walsh 2002; Di Lorenzo et al. 2003; Actis et al. 2011). On the other hand, many natural pJM1-less O1 serotype strains and other serotype strains produce and transport the chromosomally-mediated siderophore vanchrobactin (Balado et al. 2006, 2008, 2009; Soengas et al. 2006). It has been reported that *V. anguillarum* serotype O1 strain carrying the pJM1-type plasmid always produces only anguibactin but not vanchrobactin (Lemos et al. 1988). Our previous work unveiled that *V. anguillarum* 775 (pJM1) does not produce vanchrobactin due to the interruption of the *vabF* vanchrobactin biosynthesis gene by a transposon found in the pJM1 plasmid. Removal of the transposon caused the recovery of the vanchrobactin production (Naka et al. 2008) in an isogenic *V. anguillarum* 775 (pJM1) derivative. However, vanchrobactin activity was not detected from the strain that produces both anguibactin and vanchrobactin due to the competition for iron between two siderophores as anguibactin has higher iron affinity than vanchrobactin (Naka et al. 2008). Furthermore, the interruption of the *vabF* gene by the transposon was commonly found in the pJM1-carrying strains isolated from different geographical origins (Naka et al. 2008). Based on these findings, we hypothesized that the iron uptake phenotype of *V. anguillarum* 775 (pJM1) may have evolved from an ancestor that acquired the pJM1-encoded anguibactin-mediated system. This system could have a higher iron affinity than the vanchrobactin-mediated system encoded in the ancestor's chromosome (Naka et al. 2008). Although the anguibactin system has been extensively studied by our group, the cluster carrying anguibactin biosynthesis and transport genes has been only identified on the pJM1-type plasmid in *V. anguillarum* serotype O1 strains.

In this study, we show that an anguibactin gene cluster similar to that described in the *V. anguillarum* pJM1 plasmid is located on the chromosome of *Vibrio harveyi*. This organism is a marine bioluminescent bacterium ubiquitously found in seawater, mainly in the tropical regions, that is a pathogen of many marine vertebrate and invertebrate species (Thompson et al. 2004; Austin and Zhang 2006; Owens and Busico-Salcedo 2006). We also demonstrate that *V. harveyi* HY01 *angR* and *fatA* orthologs are essential for anguibactin biosynthesis and uptake, respectively. Our results suggest a possible evolutionary origin of the anguibactin system in these two *Vibrio* species strains.

## Materials and Methods

### Strains and growth conditions

Strains and plasmids used in this study are listed in Table 1. Primers used in this study are shown in Table S1. Although some strains are proposed to change species name from *V. harveyi* to *Vibrio campbellii* or vice versa based on increasing genomic data, we decide to keep the original designation pending official taxonomic revision.

**Table 1:** Strains and plasmids used in this study

Strains and plasmids	Characteristics	Reference or source
*Vibrio anguillarum* strains		
775 (pJM1)	Wild type, Washington (serotype O1, pJM1)	Crosa (1980)
775 (pJM1)-pMMB	775 (pJM1) harboring pMMB208	Naka et al. (2008)
96F-pMMB	Vanchrobactin producer (serotype O1, plasmidless) harboring pMMB208	Naka et al. (2008)
HNVA-8	CC9-16Δ*fvtA*Δ*fetA* (anguibactin indicator strain)	Naka and Crosa (2012)
*Vibrio harveyi* strains		
ATCC BAA-1116	Marine (Ocean) isolate	Lin et al. (2010)
HY01	Dead, luminescing shrimp isolate	Lin et al. (2010)
HNVH-1	HY01Δ*angR*	This study
HNVH-2	HY01Δ*angR*Δ*fatA*	This study
CAIM 148	Diseased shrimp (*Penaeus* sp.) hemolymph isolate	Lin et al. (2010)
CAIM 513T	Dead, luminescing amphipod (*Talorchestia* sp.) isolate *V. harveyi* type strain ATCC 14126	Lin et al. (2010)
CAIM 1075	Oyster (*Crassostrea gigas*) isolate	Lin et al. (2010)
CAIM 1766	Sea horse (*Hippocampus ingens*) liver isolate	Lin et al. (2010)
CAIM 1792	Diseased shrimp (*Litopenaeus vannamei*) lesion isolate	Lin et al. (2010)
*Vibrio campbellii* strains		
42A	Healthy coral (*Mussismilia hispida*) isolate	Lin et al. (2010)
CAIM 115	Shrimp (*Litopenaeus* sp.) hemolymph isolate	Lin et al. (2010)
CAIM 198	Shrimp (*Litopenaeus* sp.) hepatopancreas isolate	Lin et al. (2010)
CAIM 519T	Seawater isolate *V. campbellii* type strain ATCC 25920	Lin et al. (2010)
CAIM 1500	Snapper (*Lutjanus guttatus*) liver isolate	Lin et al. (2010)
*Escherichia coli* strains		
DH5α	F^−^, ϕ80lacZΔM15, endA1, recA1, hsdR17, (r_K_^−^m_K_^+^), supE44, thi-1, gyrA96, relA1, Δ(lacZYA-argF)U169, λ^−^	Laboratory stock
S17-1*λpir*	λ-*pir* lysogen; *thi pro hsdR hsdM*+*recA* RP4 2-Tc::Mu-Km::Tn*7* (Tp^r^ Sm^r^)	Simon et al. (1983)
Plasmids		
pGEM-T Easy	A vector for the cloning of PCR products with blue/white screening, Ap^r^	Promega
pBluescript II	Cloning vector, Amp^r^	Stratagene
pBluescript-Km	Cloning vector, Km^r^	This study
pDM4	Suicide plasmid sacB gene, R6K origin, Cm^r^	Milton et al. (1996)
pHN11	pDM4 harboring Δ*angR* of *V. harveyi* HY01	This study
pHN12	pDM4 harboring Δ*fatA* of *V. harveyi* HY01	This study
pMMB208	A broad-host-range expression vector; Cm^r^ *IncQ lacI*q *Ptac*; polylinker from M 13mp19	Morales et al. (1991)
pHN13	pMMB208 harboring *V. harveyi angR*	This study
pHN14	pMMB208 harboring *V. harveyi fatA*	This study

ATCC and CAIM strains were obtained from American Type Culture Collection (http://www.atcc.org) and Collection of Aquatic Important Microorganisms (http://www.ciad.mx/caim), respectively.

*Vibrio harveyi* strains were grown in Luria Marine (LM) medium containing LB broth (Difco, Sparks, MD) and 1.5% NaCl or in AB medium (Taga and Xavier 2011) at 30°C. Thiosulfate-citrate-bile salts-sucrose (TCBS) agar (Difco) was used as a selective medium for *V. harveyi* to counterselect *Escherichia coli*. *Escherichia coli* strains were cultured in LB broth or LB agar (LB broth supplemented with 1.5% agar) at 37°C. When needed, antibiotics were added to the medium in the following concentrations: for *V. harveyi* or *V. anguillarum*, chloramphenicol (Cm) 10 μg/mL; for *E. coli*, ampicillin 100 μg/mL, Cm 30 μg/mL.

### Generation of mutants and complementation

To construct Δ*angR* in *V. harveyi* HY01, the upstream and downstream regions of the target genes were PCR amplified using HY01*angR*–mut-up-*Sal*I-F and HY01*angR*–mut-up-*Sma*I-R, and HY01*angR*–mut-down-*Sma*I-F and HY01*angR*–mut-down-*Spe*I R primers, respectively. Both fragments were independently cloned into the pGEM-T Easy vector and subsequently subcloned into pBluescript-Km using restriction enzymes that recognize the recognition sites added in both ends of the primers. pBluescript-Km was generated by inserting the Km resistance DNA fragment obtained by *Sma*I digestion of pBlue-Km-*Sma*I (Naka et al. 2012) into the *Sca*I site of pBluescript II (Stratagene, La Jolla, CA). To construct the Δ*angR*Δ*fatA* double mutant in *V. harveyi* HY01, the PCR-amplified DNA fragments obtained using HY01*angRfatA–*mut-up-*Xho*I-F and HY01*angRfatA–*mut-up-*Sma*I-R primers were cloned into pGEM-T Easy and subsequently subcloned into pBluescript-Km together with the DNA fragment obtained using HY01*angR–*mut-down-*Sma*I-F and HY01*angR–*mut-down-*Spe*I-R.

The deletion fragments were subcloned into the suicide vector pDM4 (Milton et al. 1996) and the plasmids were maintained into *E. coli* S17-1 λ *pir*. The pDM4 derivatives thus constructed were conjugated into *V. harveyi* strains, selecting the exconjugants on TCBS plus Cm. Selected exconjugants were plated on LM plates containing 20% sucrose to isolate the proper *V. harveyi* deletion derivatives, which were screened by colony PCR using primers constructed outside (HY01*angR*–mut-up-*Sal*I-F and HY01*angR*–mut-down-*Spe*I R to check Δ*angR* and HY01*angRfatA–*mut-up-*Xho*I-F and HY01*angR*–mut-down-*Spe*I R to check Δ*angRfatA*) and inside (HY01*angR*-inter-F and HY01*angR*-inter-R to check Δ*angR* and HY01*fatA*-inter-F and HY01*fatA*-inter-R to check Δ*fatA*) of the target genes. The size of the PCR fragments obtained from the mutants using external primers was shorter than those from the wild type, and we did not observe any PCR products when internal primers were used for colony PCR in the mutants, while the wild-type positive control showed clear PCR products (data not shown).

To complement the mutants, the wild-type genes including their Shine–Dalgarno sequences were PCR amplified using primers containing restriction enzyme sites such as HY01*angR*-com-*Pst*I-F and HY01*angR*-com-*EcoR*I to complement Δ*angR*, and HY01*fatA*-com-*Sph*I-F and HY01*fatA*-com-*Xba*I-R to complement Δ*fatA*. The PCR fragments thus obtained were digested with restriction enzymes, and cloned into pMMB208 digested with the corresponding restriction enzymes (Morales et al. 1991). The pMMB208 derivatives were then conjugated into *V. harveyi* strains as described before.

### Siderophore cross-feeding bioassays

Cross-feeding assays to test the ferric-siderophore utilization were performed as previously described (Tolmasky et al. 1988). To assess anguibactin production by *V. harveyi* derivatives, *V. anguillarum* strain CC9-16Δ*fvtA*Δ*fetA* (Table 1) was used as an indicator strain of ferric-anguibactin transport (Naka and Crosa 2012). *Vibrio harveyi* HY01 derivatives were used as indicator strains to test their ability to transport ferric-anguibactin. *Vibrio anguillarum* and *V. harveyi* strains were grown overnight in AB broth at 25°C. Both 2× AB broth and 1.4% agarose (or 2% agarose) were separately prepared and autoclaved, and ethylenediamine-di-(*o*-hydroxyphenylacetic) acid (EDDA) for the *V. anguillarum* indicator strain or 2,2′-dipyridyl (DIP) for the *V. harveyi* indicator strains (and IPTG [1 mmol/L] and Cm [10 μg/mL] as needed) were added to 2× AB broth. Iron limitation conditions were obtained using EDDA for *V. anguillarum* and DIP for *V. harveyi*. This last bacterium needed very high concentrations of EDDA in the media to achieve distinct growth inhibition, possibly due to the different cell penetration of the two compounds (Chart et al. 1986). The supplemented AB broth was mixed 50:50 with 1.4% agarose (or 2% agarose). Overnight culture of the indicator strains (5 μL/mL) was added to the medium adjusted to approximately 40°C. After solidification, iron sources, the bacteria-producing siderophore, or purified siderophores were spotted on the plates. The plates were incubated at 25°C; growth halos around the spots were monitored and recorded after overnight incubation.

### Extraction of large plasmids

Plasmids of *V. harveyi, V. anguillarum,* and *Sinorhizobium meliloti* were extracted using the modified Eckhardt method described by Hynes et al. (1985) with a slight modification (in-gel lysis method). Briefly, 0.7% horizontal agarose gel was prepared using 1× Tris Borate (TB) buffer. After solidification, the material between the comb and the negative electrode end of the gel was removed, and the empty space was filled with 0.4% agarose in TB buffer with addition of 1% SDS. Overnight cultures were diluted in fresh media; LM for vibrios and TY for *S. meliloti*, and incubated until OD_600_ reached ∼0.3. Then, 200 μL of culture was mixed with 200 μL of 0.02% sarkosyl dissolved in TE buffer. After gentle inversion, the samples were centrifuged at 17,000*g* for 2 min, the culture supernatant was carefully removed and the bacterial cells were resuspended in 20 μL of lysis solution (20% sucrose, 10^5^ units/100 mL lysozyme and 10^2^ units/100 mL RNase in TE buffer). The samples were then loaded on the gel, and the electrophoresis was performed at 20 V for 20 min followed by 120 V for 3.5 h. After electrophoresis, the gel was stained with GelRed (Biotium Inc., Hayward, CA) overnight, and the image was captured by the Gel Logic 100 Imaging System (Eastman Kodak Co., Rochester, NY).

### Purification and analysis of siderophore anguibactin

Anguibactin was purified from *V. harveyi* strain HY01 using the method described previously (Actis et al. 1986). All glassware used for anguibactin purification was washed with 0.1 mol/L HCl prior to the purification to exclude iron. The purified anguibactin was checked by bioassay as well as high-performance liquid chromatography (HPLC), and nominal masses of the ion species (*m/z*) of the purified siderophore were determined by mass spectrometry at the BioAnalytical Shared Resource Core Facility at OHSU using a Thermo Electron LCQ Advantage ion trap mass spectrometer equipped with an electrospray ionization source. The full electrospray ionization-mass spectra and those with collision energy 30% (MS2) were both acquired in a positive mode using as mobile phase 1:1 methanol:water for the full scan and 0.1% formic acid for MS2.

### Extraction and analysis of outer membrane fractions

*Vibrio harveyi* strains were grown in iron rich (30 mL AB plus 10 μg/mL ferric ammonium citrate [FAC]) and iron limiting (30 mL AB broth plus 30 μmol/L DIP) until exponential phase (OD_600_ ∼0.3). Outer membrane fractions of *V. harveyi* strains were extracted by using sodium lauroyl sarcosinate (sarkosyl) as described before (Naka and Crosa 2012). Briefly, *V. anguillarum* was grown in CM9 medium until late-exponential phase. Bacteria were harvested, and pellets were resuspended into 10 mmol/L Tris–HCl (pH 7.6). The bacterial cells were broken by sonication, and cell debris was removed by centrifugation. The supernatant was transferred to another tube, and the total membranes were collected by centrifuging the tubes at 20,000*g* for 60 min. To extract outer membrane fractions, the membrane fractions were resuspended in 1.5% sodium lauroyl sarcosinate, incubated for 2 h at 4°C, and centrifuged at 20,000*g* for 1 h at 4°C. Extracted outer membrane fractions were resuspended into 30 μL of distilled water, and 5 μL of samples was subjected to SDS-PAGE using Criterion™ XT Precast Gel (10% Bis–Tris; Bio-Rad, Hercules, CA) for 5 h at 80 V at 4°C. One portion of the gel was stained with Bio-Safe™ Coomassie G-250 stain (Bio-Rad) to visualize outer membrane proteins. The other portion of the gel was used for Western blotting to detect the FatA protein using anti-*V. anguillarum* FatA polyclonal antibody. The procedure before detection of signals was performed following the protocol described before (Naka et al. 2008). Signals were detected using the Luminata Western HRP Substrates (Millipore Corp, Bedford, MA) following the manufacture's instruction, and imaged by using Image Quant LAS4000 (GE Healthcare Life Sciences, Piscataway, NJ).

## Results

### Anguibactin biosynthesis and transport cluster

**Figure 1:**
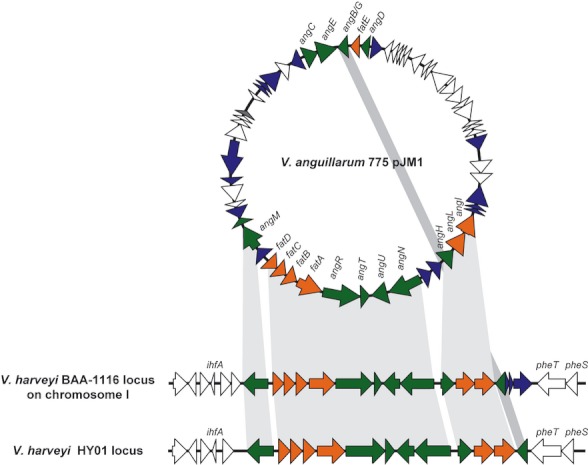
The anguibactin biosynthesis and transport genes are conserved in *Vibrio anguillarum* pJM1 and two *Vibrio harveyi* strains, BAA-1116 and HY01. Gray-shaded parts indicate that these genes are conserved between the three clusters. The green-filled arrows, orange-filled arrows, and blue-filled arrows represent the genes involved in anguibactin biosynthesis, anguibactin transport, and transposon elements, respectively.

Our BLAST search revealed that two sequenced *V. harveyi* strains, BAA-1116 and HY01, carry a homolog of the *V. anguillarum angR* gene encoded on the pJM1 plasmid. Further analysis unveiled that homologs of the majority of the genes involved in anguibactin biosynthesis and transport found in the pJM1 plasmid are located on the same genetic region in these two *V. harveyi* strains (Fig. 1), whereas other genes required for anguibactin biosynthesis are found elsewhere on the chromosome. Comparative analyses of the predicted products of the pJM1 genes with those of cognate genes present in *V. harveyi* showed that they have 63–84% identity and 77–93% similarity at the amino acid level depending on the gene (Table 2), while the products of the cognates genes present in the two different *V. harveyi* strains showed 92–99% identity and 97–100% similarity (Table 3). The predicted anguibactin biosynthesis and transport genes found in the chromosome of these two *V. harveyi* strains are found between *ihfA* (*himA*), which encodes the integration host factor alpha subunit, and *pheST,* which encodes a phenylalanine tRNA synthetase (Mechulam et al. 1985; Brown 2001; Fig. 1 and Table 3). Three genes potentially encoding transposases were located in the anguibactin cluster of strain BAA-1116 but not of strain HY01. It is of interest that highly related homologs of these genes were found on the pJM1 plasmid (two loci, ORF16-18 and ORF46-48, with three genes each annotated as *orf1-3* ISVme). Furthermore, these transposase genes were frequently found in the BAA-1116 chromosome and plasmid, as well as in the *V. anguillarum* 775 chromosomes (data not shown). However, no homologs of these genes were detected in the HY01 draft genome sequence. Given that the anguibactin cluster was placed close to the *pheST* t-RNA locus, it could possibly be a pathogenicity island horizontally acquired during evolution. However, we did not find a clear difference in the GC content between the anguibactin cluster (BAA-1116, 44.8%: HY01, 45.0%) and the *V. harveyi* genome (BAA-1116, 45.4%: HY01, 45.6%).

**Table 2:** Comparison of genes on the pJM1 plasmid and on the *Vibrio harveyi* anguibactin locus

	*V. harveyi* HY01			*V. harveyi* BAA-1116		
		
pJM1 ORF number (gene name)	Accession number	Identity	Similarity	Accession number	Identity	Similarity
1 (*angM*)	ZP_01986345	444/706 (63%)	547/706 (77%)	VIBHAR_02109	445/706 (63%)	546/706 (77%)
2	NA	NA	NA	NA	NA	NA
3 (*fatD*)	ZP_01986358	263/314 (84%)	290/314 (92%)	VIBHAR_02108	264/314 (84%)	291/314 (93%)
4 (*fatC*)	ZP_01986350	245/317 (77%)	276/317 (87%)	VIBHAR_02107	246/317 (78%)	276/317 (87%)
5 (*fatB*)	ZP_01986380	265/324 (82%)	298/324 (92%)	VIBHAR_02106	262/324 (81%)	298/324 (92%)
6 (*fatA*)	ZP_01986352	564/725 (78%)	643/725 (89%)	VIBHAR_02105	560/725 (77%)	638/725 (88%)
7 (*angR*)	ZP_01986376	666/1046 (64%)	816/1046 (78%)	VIBHAR_02104	661/1046 (63%)	813/1046 (78%)
8 (*angT*)	ZP_01986361	159/249 (64%)	198/249 (80%)	VIBHAR_02103	127/202 (63%)	163/202 (81%)
9 (*angU*)	ZP_01986389	350/439 (80%)	387/439 (88%)	VIBHAR_02102	347/439 (79%)	386/439 (88%)
10 (*angN*)	ZP_01986387	659/952 (69%)	775/952 (81%)	VIBHAR_02101	653/952 (69%)	770/952 (81%)
11–12	NA	NA	NA	NA	NA	NA
13 (*angH*)	ZP_01986363	313/386 (81%)	351/386 (91%)	VIBHAR_02100	311/386 (81%)	350/386 (91%)
14 (*angL*)	ZP_01986390	384/536 (72%)	458/536 (85%)	VIBHAR_02099	384/536 (72%)	454/536 (85%)
15 (*angI*)	ZP_01986392	372/532 (70%)	444/532 (83%)	VIBHAR_02098	371/532 (70%)	440/532 (83%)
16–40	NA	NA	NA	NA	NA	NA
41 (*angB/G*)	ZP_01986357	228/288 (79%)	258/288 (90%)	VIBHAR_02097	204/256 (80%)	229/256 (89%)
42–59	NA	NA	NA	NA	NA	NA

NA, not applicable (no homolog in the *V. harveyi* anguibactin locus).

**Table 3:** Comparison of genes on the anguibactin locus of *Vibrio harveyi* BAA-1116 and HY01

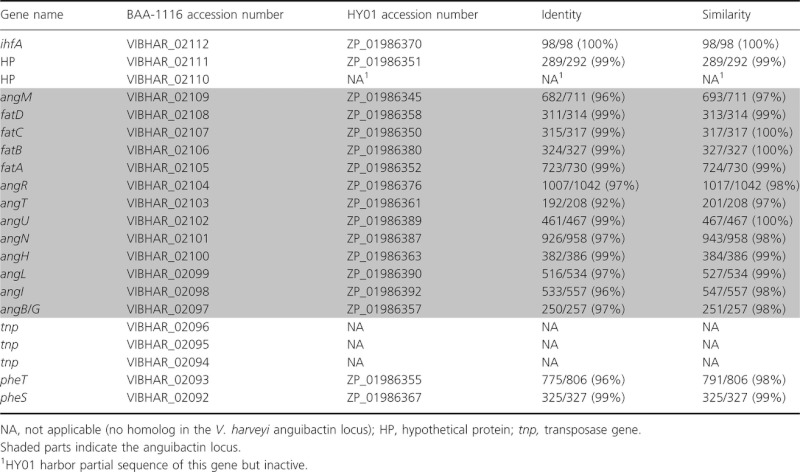

According to the whole-genome sequence data of *V. harveyi* BAA-1116 in Genbank, the anguibactin cluster is located on chromosome 1. Due to the fact that gap closure of *V. harveyi* HY01 DNA contigs has not been completed yet, we could not determine whether the anguibactin cluster is located on the chromosome of this strain. We first determined whether *V. harveyi* HY01 harbors any plasmids using the modified in-gel lysis method that can identify very large plasmids as described in Materials and Methods. The results indicate that this strain carries one plasmid with a molecular size between 8 and 13 kb estimated by gel electrophoresis using supercoiled DNA markers (Fig. S1). The cluster between *ihfA* and *pheST* harboring anguibactin locus expands >29 kb, which is larger than the size of the observed plasmid. As positive controls of this method, large plasmids were successfully detected from *V. anguillarum* 775 (pJM1) harboring the 65-kb pJM1 plasmid, *V. harveyi* BAA-1116 harboring the 89-kb pVIBHAR plasmid, and *Sinorhizobium meliloti* 102F34 harboring 100-, 150-, and 220-kb plasmids (Cook et al. 2001; Fig. S1). From these experiments, we conclude that the anguibactin cluster of strain HY01 is also located on the chromosome rather than on the plasmid.

### Anguibactin production by *V. harveyi*

**Figure 2:**
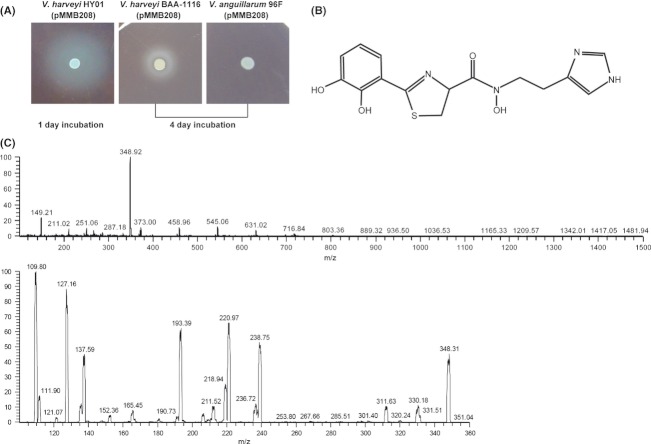
Anguibactin production from *Vibrio harveyi*. (A) Bioassay to test anguibactin production. CC9-16Δ*fvtA*Δ*fetA* was used as an anguibactin indicator strain. EDDA (40 μmol/L) and Cm (10 μg/mL) were added into AB media with the indicator strain. Five microliters of overnight culture of *V. harveyi* HY01 (pMMB208), *V. harveyi* BAA-1116 (pMMB208), and *Vibrio anguillarum* 96F (pMMB208) grown in AB broth with Cm was spotted on the plates, and incubated at 25°C. Presence of growth halos around spots was checked every 24 h. *Vibrio anguillarum* 96F (pMMB208) is a vanchrobactin producer and was used as a negative control. (B) Structure of anguibactin from *V. anguillarum* 775 (pJM1) (Jalal et al. 1989). (C) Confirmation of anguibactin biosynthesis in the *V. harveyi* HY01 strain. Positive mode electrospray ionization-mass spectra of the purified siderophores without (above) and with (below) 30% collision energy. The nominal masses (*m/z*) of the parental ion species and different fragmentation products are indicated in the spectra.

The high similarity of the *V. harveyi* anguibactin gene cluster with that found in the *V. anguillarum* 775 pJM1 plasmid suggested that *V. harveyi* BAA-1116 and HY01 likely produce anguibactin. This possibility was tested with siderophore utilization assays using *V. anguillarum* CC9-16Δ*fvtA*Δ*fetA* as a reporter strain. In *V. anguillarum*, FvtA is functional for both ferric-vanchrobactin and ferric-enterobactin transport, while FetA is involved in ferric-enterobactin transport (Balado et al. 2009; Naka and Crosa 2012). As shown in Figure 2A, CC9-16Δ*fvtA*Δ*fetA* can utilize siderophores produced by either *V. harveyi* BAA-1116 or HY01 indicating that the siderophores produced by these strains are likely anguibactin. However, the data indicated that strain HY01 produces more anguibactin than BAA-1116; a larger growth halo was produced with the supernatants from HY01 after 1 day of incubation, while a smaller halo was detected with BAA-1116 supernatants after 4 days of incubation under the same experimental conditions. Based on these observations, we decided to use HY01 for further characterization of the anguibactin-mediated system expressed by *V. harveyi*.

As the ferric-anguibactin outer membrane receptor FatA has been shown to be able to transport at least two different siderophores, anguibactin and acinetobactin (Dorsey et al. 2004), it was important to use a different approach to ensure that the siderophore is indeed anguibactin. The structure of anguibactin from *V. anguillarum* 775 (pJM1) determined previously (Jalal et al. 1989) is shown in Figure 2B. We purified the siderophore from strain HY01 and performed mass spectrometry analysis. As shown in Figure 2C, a strong peak with a mass 348.92 was detected by ElectroSpray Ionization-Mass Spectrometry (ESI-MS) in the siderophore isolated and purified from HY01 iron-limiting culture supernatants. The molecular mass of this peak is in agreement with the molecular weight of anguibactin from *V. anguillarum* 775 (pJM1) reported before (Actis et al. 1986). The nature of the siderophore produced by *V. harveyi* HY01 was further confirmed by tandem mass spectrometry (ESI-MS/MS) analysis; the fragmentation pattern produced by the siderophore isolated from HY01 iron-limiting culture supernatants was identical (within the error limits) to the pattern by anguibactin purified from *Vibrio* sp. DS40M4 (Sandy et al. 2010). Taken together, these results confirm that *V. harveyi* HY01 actually produces anguibactin.

### AngR is involved in the biosynthesis of anguibactin and is required for the growth under iron-limiting conditions

**Figure 3:**
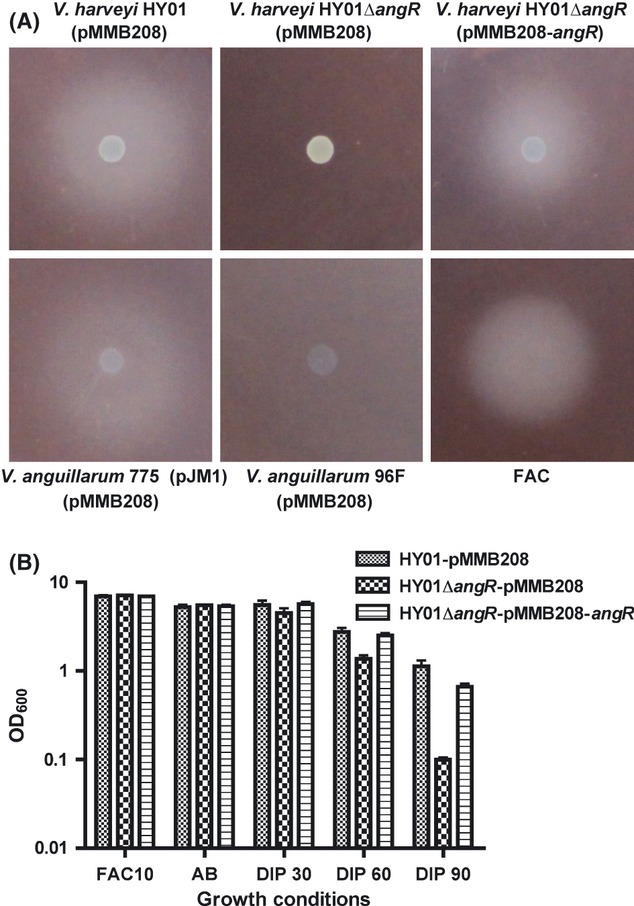
Characterization of the *angR* gene in *Vibrio harveyi* HY01. (A) The *angR* gene is essential for the anguibactin production in *V. harveyi* HY01. *Vibrio anguillarum* CC9-16Δ*fvtA*Δ*fetA* was used as an anguibactin indicator strain. EDDA (40 μmol/L) and Cm (10 μg/mL) were added into AB media with the indicator strain. Five microliters of an overnight culture of *V. harveyi* HY01 (pMMB208), *V. harveyi* HY01Δ*angR* (pMMB208), *V. harveyi* HY01Δ*angR* (pMMB208-*angR*), *Vibrio anguillarum* 775 (pJM1) (pMMB208), and *V. anguillarum* 96F (pMMB208) grown in AB broth with Cm and 1 μL of 1 mg/mL ferric ammonium citrate (FAC) were spotted on the plates and were incubated at 25°C. Presence of growth halos around spots was checked after 24-h incubation. *V*. *anguillarum* 775 (pJM1) (pMMB208) and FAC are positive controls. *Vibrio anguillarum* 96F (pMMB208) is a vanchrobactin producer and was used as a negative control. (B) Anguibactin production promotes the growth of *V. harveyi* in iron-limiting conditions. Fifty microliters of overnight culture (adjusted OD_600_ to 1) grown in 5 mL AB broth with Cm (10 μg/mL) was inoculated into AB broth containing Cm (10 μg/mL) and IPTG (1 mmol/L) (AB) or with addition of 10 μg/mL ferric ammonium citrate (FAC10), 30 μmol/L dipyridyl (DIP30), 60 μmol/L dipyridyl (DIP60), or 90 μmol/L dipyridyl (DIP90). OD_600_ was measured after 24-h incubation at 25°C. Experiments were repeated five times, and the error bars show standard deviation.

To check whether *angR* is required for the anguibactin biosynthesis in *V. harveyi* as it was previously described in *V. anguillarum* 775 (pJM1) (Wertheimer et al. 1999), we constructed and tested the *V. harveyi* HY01Δ*angR* deletion mutant. Our bioassay results (Fig. 3A) showed that this derivative did not produce any anguibactin when compared to the isogenic HY01 wild-type strain. However, production of anguibactin was restored when the *V. harveyi* HY01Δ*angR* was *trans* complemented with the wild-type *angR* allele. We also compared the growth rate of the parental HY01 strain and the isogenic *angR* mutant under different iron growth conditions (Fig. 3B). The mutation in *angR* did not affect the growth of *V. harveyi* HY01 in iron sufficient conditions, while increasing the amount of the iron chelator 2,2′-dipyridyl (DIP), which creates iron limitation in the AB medium, impaired the growth of the *angR* mutant as compared with the wild-type strain. Complementation of the mutant with the wild-type *angR* gene *in trans* restored growth to wild-type levels. From these results, we conclude that AngR is important for *V. harveyi* to produce anguibactin and survive under iron-limiting conditions.

### *Vibrio harveyi* HY01 FatA homolog is involved in the ferric-anguibactin utilization and located on the outer membrane fractions

**Figure 4:**
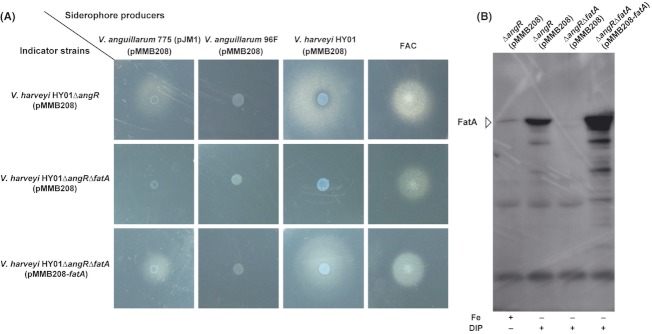
The *fatA* gene in *Vibrio harveyi* HY01 encodes the ferric-anguibactin outer membrane receptor protein. (A) The *fatA* gene is involved in the ferric-anguibactin transport in *V. harveyi* HY01. *V. harveyi* HY01Δ*angR* (pMMB208), *V. harveyi* HY01Δ*angR*Δ*fatA* (pMMB208), and *V. harveyi* HY01Δ*angR*Δ*fatA* (pMMB208-*fatA*) were used as indicator strains. DIP (100 μmol/L), IPTG (1 mmol/L), and Cm (10 μg/mL) were supplemented into AB medium with indicator strains. Five microliters of overnight culture of *V. harveyi* HY01 (pMMB208) and *Vibrio anguillarum* 775 (pJM1) (pMMB208) and 1 μL of 1 mg/mL ferric ammonium citrate (FAC) were spotted on the plates. Presence of growth halos around spots was checked after 24-h incubation at 25°C. (B) The FatA protein locates on the outer membrane of *V. harveyi*. *Vibrio harveyi* HY01Δ*angR* (pMMB208), *V. harveyi* HY01Δ*angR*Δ*fatA* (pMMB208), and *V. harveyi* HY01Δ*angR*Δ*fatA* (pMMB208-*fatA*) were grown in AB medium until exponential phase (OD_600_ ∼0.3). Outer membrane proteins were then extracted using sarkosyl as described in Materials and Methods. Western blots were performed using anti-*V. anguillarum* FatA polyclonal antibody.

In *V. anguillarum* 775 (pJM1), FatA is the outer membrane ferric-anguibactin receptor that plays an essential role in the uptake of the ferric-anguibactin complex from the external environment to the periplasmic space (Lopez and Crosa 2007). The high similarity of amino acid sequences of FatA from *V. anguillarum* 775 (pJM1) and *V. harveyi* HY01 motivated us to characterize the function and the localization of the FatA protein in *V. harveyi* HY01. To evaluate whether FatA is essential for the ferric-anguibactin utilization in *V. harveyi*, we constructed a Δ*fatA* mutant in strain HY01 and performed bioassays. Our results (Fig. 4A) show that the Δ*fatA* mutant cannot utilize ferric-anguibactin to grow in iron-limiting conditions, while the wild-type strain as well as the Δ*fatA* mutant complemented with the *fatA* gene enhances the growth by acquiring anguibactin from both *V. anguillarum* 775 (pJM1) and *V. harveyi* HY01. These results indicate that in *V. harveyi* HY01, *fatA* is essential to acquire ferric-anguibactin as an iron source.

To investigate whether FatA localizes in the outer membrane fractions of *V. harveyi* HY01 as it was previously described in *V. anguillarum* 775 (pJM1), outer membrane proteins were extracted using sodium lauroyl sarcosinate from *V. harveyi* HY01 grown in iron-rich and iron-limiting conditions, and Western blotting using anti-*V. anguillarum* FatA was performed to detect *V. harveyi* FatA (Fig. 4B). A small amount of *V. harveyi* FatA was detected when the cells were grown in iron-rich conditions, while much more *V. harveyi* FatA was observed when the cells were grown under iron limitation. Deletion of *fatA* in *V. harveyi* caused complete loss of FatA protein in the outer membrane fraction, while the *V. harveyi* Δ*fatA* complemented with the *V. harveyi fatA* showed very high levels of FatA in the outer membrane fraction. These results indicate that *V. harveyi* FatA locates on the outer membrane of *V. harveyi* HY01, and the expression of FatA is upregulated in iron-limiting conditions.

### Anguibactin production from various strains

**Figure 5:**
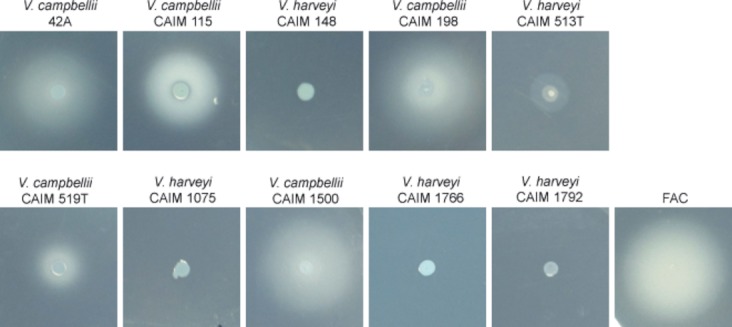
Evaluation of anguibactin production from various strains. *Vibrio anguillarum* CC9-16Δ*fvtA*Δ*fetA* was used as an anguibactin indicator strain. EDDA (40 μmol/L) was added into AB media with the indicator strain. Higher concentration of agarose (1%) was supplemented into AB media to reduce swarming of some strains. Overnight culture grown in AB broth was spotted on the plates, and the plates were incubated at 25°C. Presence of growth halos around spots was checked after 24-h incubation. Clear zone around the spot observed in *Vibrio harveyi* CAIM 513T exhibits swarming but not anguibactin production. Anguibactin production was never detected in anguibactin production negative strains shown in this figure even after incubation for several days. Experiments were repeated three times, and this figure shows a representative.

Recently, we determined the genome sequence of *V. campbellii* DS40M4 that produces anguibactin (Dias et al. 2012), and our DNA sequence analysis showed that this strain also carries an almost identical anguibactin locus to the one found in the two *V. harveyi* strains used in this work. However, we did not find the anguibactin cluster in the draft genome sequence of *V. harveyi* CAIM 1792 (Espinoza-Valles et al. 2012). Lin et al. (2010) proposed that *V. harveyi* BAA-1116 and HY01 should be classified as *V. campbellii* based on microarray-based comparative genomic hybridization (CGH) and multilocus sequence analyses (MLSA). According to this paper, *V. harveyi* CAIM 1792 is the only *V. harveyi* strain in which whole-genome sequence data are available in a public database. We checked anguibactin production from five strains of *V. harveyi* and five strains of *V. campbellii* as classified by Lin et al. (2010). Our results showed that strains classified as *V. harveyi* do not produce anguibactin, while strains classified as *V. campbellii* produce anguibactin when CGH and MLSA were applied to classify bacterial species (Fig. 5).

## Discussion

Many *V. anguillarum* O1 serotype strains carry the 65-kb pJM1-type plasmid encoding the siderophore anguibactin biosynthesis and transport genes. The anguibactin system is essential for *V. anguillarum* to survive under iron-limiting conditions including those found in their host organisms. Thus, the curing of the pJM1 plasmid causes growth defect in iron-deficient conditions. Our previous work suggested that a *V. anguillarum* pJM1-less strain acquired the pJM1 plasmid during evolution, and gained the very high-affinity iron transport anguibactin system, following inactivation of the original chromosomally-mediated siderophore vanchrobactin (Naka et al. 2008). Anguibactin production has been found only in *V. anguillarum* serotype O1 strains harboring pJM1-like plasmids, and in the *Vibrio* sp. DS40M4 isolate (later named as *V. campbellii* DS40M4) so far (Sandy et al. 2010).

In this work, we report that two *V. harveyi* strains, BAA-1116 and HY01, produce and utilize anguibactin. For the first time, we identified and genetically characterized a *V. harveyi* gene cluster carrying the majority of homologous genes encoding anguibactin biosynthesis and transport proteins found in the pJM1 plasmid of *V. anguillarum* 775 (pJM1). Homologs of the pJM1 encoding anguibactin biosynthesis genes such as *angC*, *angE,* and *dhap* involved in 2,3-dihydroxybenzoic acid (DHBA) biosynthesis and *angD*, encoding a phosphopantetheinyl transferase, were not found in these *V. harveyi* anguibactin clusters. However, in the case of *V. anguillarum*, functional homologs of those genes are located on the chromosome locus associated with another siderophore biosynthesis and transport system (Alice et al. 2005; Naka et al. 2008). Interestingly, in the human pathogen *Acinetobacter baumannii, entA,* which is essential for DHBA biosynthesis, is located outside of the acinetobactin gene cluster that otherwise contains all the genes needed for acinetobactin biosynthesis, export, and transport (Penwell et al. 2012). It appears that in *V. harveyi,* we can find a similar situation with the homologs of *angC*, *angE,* and *angD*. These genes exist in a different location on the chromosome (AngC homolog [BAA-1116: VIBHAR_01398 49% identity and 66% similarity, HY01: A1Q_1381 50% identity and 66% similarity], AngE homolog [BAA-1116: VIBHAR_01399 55% identity 70% similarity, HY01: A1Q_1380 56% identity and 70% similarity], and AngD homolog [BAA-1116: VIBHAR_01403 33% identity and 57% similarity, HY01: A1Q_1376 33% identity and 57% similarity]) and those homologs are included in the same cluster potentially involved in siderophore biosynthesis and transport.

Characterization of two genes, *angR* and *fatA* located on the *V. harveyi* HY01 chromosome locus, unveiled that these genes are involved in anguibactin biosynthesis and transport, respectively, as is the case of *V. anguillarum*. These findings suggest that the anguibactin cluster found in the pJM1 plasmid of *V. anguillarum* serotype O1 strains could have originated from the chromosome locus found in the *V. harveyi* (or *V. campbellii*). Nonetheless, we cannot exclude the possibility that the *V. harveyi* chromosomally-mediated anguibactin locus was acquired from the pJM1-type plasmid.

Our bioassay results showed that *V. campbellii* rather than *V. harveyi* produces anguibactin following the definition proposed by Lin et al. (2010) using microarray-based CGH and MLSA. As we still need to wait for the conclusion of the “*V. harveyi* or *V. campbellii*” debate, in this study we chose to keep the original designation pending official taxonomic revision. It is of interest that only the strains classified as *V. campbellii* by CGH and MLSA produce and utilize anguibactin.

In summary, for the first time, we identified and characterized the chromosomally encoded anguibactin cluster from *V. harveyi*. This finding indicates that the anguibactin cluster found in the pJM1 plasmid of *V. anguillarum* serotype O1 strains could possibly have been acquired from the chromosome locus found in *V. harveyi*.

## Supporting Information

**Figure S1.** The *V. harveyi* HY01 anguibactin cluster is not located on the plasmid. Presence of plasmids from *V. harveyi* HY01 was examined using “In gel lysis method” as described in Materials and Methods. *Sinorhizobium meliloti* 102F34 containing 100, 150 and 220 kb plasmids (Cook et al. 2001), *V. harveyi* BAA-1116 containing the 89 kb pVIBHAR plasmid, *V. anguillarum* 775(pJM1) containing the 65 kb pJM1 plasmid were used as controls. Supercoiled DNA Marker Set (Epicentre, Madison, WI) were used to estimate the size of supercoiled plasmid DNA. Experiments were repeated three times, and the picture is a representative.

**Table S1.** Primers used in this study.
