# Supplementary material for: The anguibactin biosynthesis and transport genes are encoded in the chromosome of Vibrio harveyi: a possible evolutionary origin for the pJM1 plasmid–encoded system of Vibrio anguillarum?
Source: Microbiologyopen. 2013 Jan 18;2(1):182–94. doi: 10.1002/mbo3.65 (PMC3584223; doi:10.1002/mbo3.65)
Supplement: Supplementary file 1 [file mbo30002-0182-SD1.docx]

| **Table S1. Primers used in this study** | | |
| --- | --- | --- |
| Primer name | Nucleotide sequence (5’ to 3’) | Objective |
| HY01*angR*–mut-up-*Sal*I-F | GTCGACACCTTCATCCGACACCCTTTTAGC | To construct and verify ∆*angR* |
| HY01*angR*–mut-up-*Sma*I-R | CCCGGGATGGTTCGCTTTGTTTCATTGTG | To construct ∆*angR* |
| HY01*angR*–mut-down-*Sma*I-F | CCCGGGCGTGATCATGAGTCCTTTAG | To construct ∆*angR* or ∆*angR*∆*fatA* |
| HY01*angR*–mut-down-*Spe*I-R | ACTAGTTATACCATCGCGGATGTCTTTG | To construct and verify ∆*angR* or ∆*angR*∆*fatA* |
| HY01*angRfatA*-mut-up-*Xho*I-F | CTCGAGTCGCTTATTCGACCTCCAGATC | To construct and verify ∆*angR*∆*fatA* |
| HY01*angRfatA*-mut-up-*Sma*I-R | CTCGAGTCGCTTATTCGACCTCCAGATC | To construct ∆*angR*∆*fatA* |
| HY01*angR*-inter-F | TCACGCTCCTTGAGGTGTTGTG | To verify ∆*angR* |
| HY01*angR*-inter-R | GAGCTCTGGATTGGCGGTGACG | To verify ∆*angR* |
| HY01*fatA*-inter-F | GAGGATTTAACCTCGATGTCG | To verify ∆*fatA* |
| HY01*fatA*-inter-R | CTGACGTACGTTTACTGTCTAAC | To verify ∆*fatA* |
| HY01angR-com-*Pst*I-F | CTGCAGCTCTTACGCCCTTAGCCATCTGAC | To complement ∆*angR* |
| HY01angR-com-*EcoR*I-R | GAATTCTCACGCTCCTTGAGGTGTTGTG | To complement ∆*angR* |
| HY01*fatA*-com-*Sph*I-F | GCATGCAACGGTCAAAGCCGTCTCTGG | To complement ∆*fatA* |
| HY01*fatA*-com-*Xba*I-R | TCTAGACTAGAAATCCGCAGTGGCTG | To complement ∆*fatA* |

**
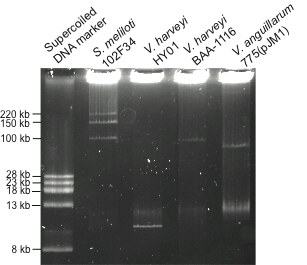
**

**Fig. S1.** The *V. harveyi* HY01 anguibactin cluster is not located on the plasmid.

Presence of plasmids from *V. harveyi* HY01 was examined using “In gel lysis method” as described in the Material and Methods section. *Sino*rhizobium meliloti 102F34 containing 100, 150 and 220 kb plasmids (Cook et al., 2001), *V. harveyi* BAA-1116 containing the 89 kb pVIBHAR plasmid, *V. anguillarum* 775(pJM1) containing the 65 kb pJM1 plasmid were used as controls. Supercoiled DNA Marker Set (Epicentre, Madison, WI) were used to estimate the size of supercoiled plasmid DNA. Experiments were repeated three times, and the picture is a representative.
